# Titanium Corrosion in Peri-Implantitis

**DOI:** 10.3390/ma13235488

**Published:** 2020-12-02

**Authors:** Mailis D. Soler, Shu-Min Hsu, Chaker Fares, Fan Ren, Renita J. Jenkins, Luiz Gonzaga, Arthur E. Clark, Edgar O’Neill, Dan Neal, Josephine F. Esquivel-Upshaw

**Affiliations:** 1 Division of Prosthodontics, Department of Restorative Dental Sciences, College of Dentistry, University of Florida, Gainesville, FL 32610, USA; MSoler@dental.ufl.edu (M.D.S.); shuminhsu@ufl.edu (S.-M.H.); BCLARK@dental.ufl.edu (A.E.C.); EONEILL@dental.ufl.edu (E.O.); 2 Department of Chemical Engineering, University of Florida, Gainesville, FL 32610, USA; c.fares@ufl.edu (C.F.); fren@che.ufl.edu (F.R.); 3 Dental Clinical Research Unit, University of Florida, Gainesville, FL 32610, USA; RJenkins@dental.ufl.edu; 4 Center for Implant Dentistry, College of Dentistry, University of Florida, Gainesville, FL 32610, USA; lgonzaga@dental.ufl.edu; 5 Department of Neurosurgery, University of Florida, Gainesville, FL 32610, USA; dneal@ufl.edu

**Keywords:** peri-implantitis, titanium particles, biopsy

## Abstract

Titanium (Ti) corrodes clinically in the presence of bacteria. We investigated this phenomenon as a function of Ti particles found in biopsied tissues around peri-implantitis sites and surface roughness of failed Ti implants. Tissue biopsies were surgically collected from peri-implantitis sites, processed, and embedded in resin. The resin-embedded samples were hand trimmed to the region of interest and semi-thick (500 nm) sections were collected onto coverslips. One section was toluidine blue post-stained as a reference. The remainder sections were left unstained for energy-dispersive X-ray spectroscopy (EDX) analysis. Processed samples were examined under scanning electron microscopy (SEM) and EDX. Corresponding failed implants were also removed and examined under SEM and EDX. Five out of eight biopsied samples demonstrated the presence of Ti particles in the soft tissue, suggesting the true rate among all failures was between 24.5% and 91.5% (the lower bound of a 95% confidence interval for the true rate of Ti presence). SEM analysis of failed implant bodies also indicated changes in surface morphology and appeared less detailed with decreased weight percent of Ti on the surface of the failed implants. In conclusion, Ti particles were noted in 5/8 biopsied samples. Surface morphologies were smoother in failed implants compared with the reference implant.

## 1. Introduction

Peri-implantitis is an inflammatory disease that affects the peri-implant mucosa and the supporting bone [1,2]. A systematic report by Derks and Tomasi [3] reported a weighted mean prevalence of peri-implantitis of 22% based on a mix of reports in the literature with inconsistencies in case definitions and a large variation in disease prevalence. They emphasized the importance of conducting disease prevalence studies on large, randomly selected patient samples with adequate radiographic assessment of peri-implant bone loss.

Derks and co-workers [4] conducted a combined retrospective analysis and cross-sectional clinical and radiologic examination on 588 patients and 2277 implants that were followed for nine years. They reported that 45% of all patients presented with peri-implantitis (defined as bleeding on probing/suppuration and bone loss >0.5 mm); and 14.5% of all patients presented with moderate/severe peri-implantitis (defined as bleeding on probing/suppuration and bone loss >2 mm).

The available literature about the pathogens associated with peri-implantitis provides conflicting evidence. On one hand, next-generation sequencing studies by Kumar et al. [5] and Maruyama et al. [6] reported that the peri-implant and the periodontal microbiomes are significantly different from each other both in health and disease. In contrast, using similar next-generation sequencing methods for analysis, Sanz-Martin and co-workers [7] found that peri-implantitis sites were heavily colonized by red complex species as well as newly proposed pathogens, concluding that the peri-implantitis microbiome is commensal-depleted and pathogen-enriched, and that these peri-implantitis sites harbor pathogens associated with periodontal disease. Discrepancies in findings may be related to the use of different methods of sample collection and sequencing platforms [7]. This complicates the choice of treatment to effectively treat peri-implantitis.

Understanding comorbid conditions and factors associated with peri-implantitis further complicates the treatment of the disease. The 2017 World Workshop on Peri-Implantitis [8] conducted an extensive review of the literature to provide evidence-based conclusions on risk factors for peri-implantitis. They determined that (1) both a history of periodontitis and poor plaque control constituted strong risk factors for peri-implantitis; (2) there is no conclusive evidence that smoking and diabetes are risk factors for peri-implantitis; excess cement, genetic factors, and iatrogenic factors constitute potential risk factors for the disease; (3) there is limited evidence that keratinized mucosa and systemic conditions constitute risk factors for peri-implantitis; (4) there is no evidence that occlusal overload is a risk factor for peri-implantitis; and, lastly, (5) the role of titanium or metal particles in the pathogenesis of the disease cannot be evaluated with the currently available evidence. Our study focused particularly on learning about the association of titanium particles with peri-implantitis.

Titanium oxide is used to manufacture everyday products, such as foods, cosmetics, toothpastes, and medicines. Thus, titanium particles are frequently detected in healthy and diseased peri-implant mucosa, as well as in patients without titanium implants [9]. However, the concentrations of titanium particles have been found to be higher in the mucosa of patients with implants than in the gingiva of patients without implants [10,11,12], and even higher in patients with implants suffering from peri-implantitis [13,14].

Several factors can facilitate implant corrosion in the oral cavity, such as pH, bacteria, chemicals, and other contaminants [15,16,17,18,19]. Bacteria produce acidic toxins and acidify the surrounding environment. This can cause disruption or dissolution of the titanium oxide layer [17]. In addition, commercial toothpastes and gels are used to prevent dental caries by incorporating fluorides. However, the fluorides can dissolve the titanium oxide and weaken the protection of oxide from corrosion [19,20]. The unprotected titanium is exposed and corrodes in the presence of acid. Mechanical conditions (loading and mastication) also create microcrack and oxide layer fractures [15]. Combining chemical, biological, and mechanical wear further aggravates the titanium corrosion [15,17].

Implant corrosion products found in peri-implant mucosa can be also the result of mechanical wear during replacement of restorative components and function, and is exacerbated by exposure to chemical agents and interaction of the implant surface with byproducts of the colonizing biofilm and inflammatory cells [9]. In a review of the literature on the effects of implant corrosion products on peri-implant mucosa, Oliveira and co-workers [21] reported that, in the presence of titanium particles, osteoclast activity is activated, the number of macrophages in the site increases, and there is a higher rate of mutations in human cells cultured in titanium-based nanoparticles. Titanium particles derived from dental implants have also been found to accumulate systemically and have potential for toxic and hypersensitive effects [9,22]. Histological findings have confirmed the presence of larger lesions compared with periodontal disease lesions, and an overabundance of inflammatory cells such as neutrophils, macrophages, and plasma cells [23]. In addition, TNF-α and IL-1β, dominant osteoclast activating cytokines, were abundant in peri-implantitis sites [24]. The pathogenesis of this disease is theorized to be an initiating reaction that leads to a shift in increased pathogenic bacteria, which in turn leads to an activation of an inflammatory mechanism.

Our study aimed to research the association between implant corrosion products and peri-implantitis. We tested the hypothesis that titanium corrodes clinically in the presence of bacteria as a function of titanium particles found in tissue and the surface roughness of failed titanium implants. The results provided in this paper are based on our pilot study.

## 2. Materials and Methods

### 2.1. Clinical Study

Institutional Review Board approval was obtained through the University of Florida IRB (IRB201703331, Gainesville, FL, USA). Informed consent was obtained for each patient prior to screening. Peri-implantitis patients were identified in the Graduate Periodontics, Graduate Prosthodontics, and Implant Center Clinics from January 2018 to December 2019. Peri-implantitis was defined according to the definition of the American Academy of Periodontology. Severe or hopeless implants, which sustained excessive bone loss and displayed mobility and/or chronic infection, were biopsied and subsequently removed. Soft tissue biopsies were stored in formalin. Implants were subsequently removed using either the reverse torque technique or an alternative technique as appropriate, disinfected in CaviCide, and stored in deionized (DI) distilled water.

Prior to implant explantation, the implant sites were clinically evaluated. Data recorded included location in the mouth, implant brand, time of survival, status of the restoration, estimated percentage of radiographic bone loss, presence of erythema, mobility, bleeding on probing, suppuration, and presence of metal restorations in the same quadrant. The periodontal condition and recent history of scaling and root planning (SRP) of the subjects were also noted. The information about the patients’ demographics and comorbidities were not evaluated in this study due to the small sample size.

### 2.2. Soft Tissue Biopsy Preparation

The retrieved soft tissue biopsies were immersed in formalin after collection from the patients. The samples were sliced into 4–6 sections, depending on the thickness of the samples, and were examined under an optical microscope (magnification 1000×) to identify areas with possible titanium particles. Once these sections were determined to possibly contain Ti particles, they were processed for viewing under the scanning electron microscopy (SEM, FEI Nova 430, Hillsboro, OR, USA). The tissues were further dissected to 1 mm^3^ and fixed with 4% paraformaldehyde and 2.5% glutaraldehyde in 0.1 M sodium cacodylate containing 2 mM MgCl_2_, 1 mM CaCl_2_, and 0.25% NaCl, pH 7.23. Following washing with 0.1 M sodium cacodylate, tissues were washed with deionized water twice. Tissues were dehydrated through a graded series of ethanol solutions (25–100%), with 5% increments, one time each dilution; this was then followed by substitution with 100% acetone twice. Dehydrated samples were infiltrated with anhydrous acetone/Araldite-502/Embed-812 epoxy resin containing Z6040 embedding primer (Electron Microscopy Sciences (EMS), Hatfield, PA, USA) mixed 3:1, 1:1, 1:3, once each dilution. This was followed by 100% epoxy resin infiltrations thrice and final infiltration overnight on a rotary shaker. Polymerization was performed by incubation at 60 °C for 3 days. The resin-embedded samples were hand trimmed to the region of interest and semi-thick (500 nm) sections were collected onto Nunc Thermanox coverslips (Thermo Fischer Scientific, Rochester, NY, USA). One section was post-stained with toluidine blue as a reference. The remainder of the sections were left unstained for SEM and energy-dispersive X-ray spectroscopy (EDX) analysis. Tissues were processed with the aid of a (MW) Pelco BioWave laboratory microwave (Ted Pella, Redding, CA, USA) (Table 1).

### 2.3. SEM and EDX Analysis of Implant Bodies

The morphologies of the failed implants were examined under scanning electron microscopy at 1000× magnification (SEM). Samples were cleaned using UV ozone to remove the organic matter and/or contamination from the surface prior to SEM examination. The images were obtained at 10 kV for reference and failed implants. After, the surface compositions were analyzed using energy-dispersive X-ray spectroscopy (EDX) at 150× to determine if there was Ti presence.

### 2.4. Statistical Analysis

The presence of Ti particles was treated as a dichotomous variable, and a 95% confidence interval for the true population rate of Ti presence was calculated using the conservative method of Clopper–Pearson [25]. All analyses were performed using the R statistical software package (V.4.0.2, The R Foundation for Statistical Computing, Vienna, Austria).

## 3. Results

Eight patients with dental implants clinically diagnosed as failing were recruited for the study. The most critical clinical signs that determined failure and need for removal of the implants were mobility and chronic infection that did not improve with treatment. Most of the samples displayed bone loss radiographically. A list of all the samples and their clinical descriptive data are presented in Table 2. Seven out of eight failed implants were retrieved from the posterior jaws. Five specimens were collected from the mandible, and three from the maxilla. The specimens were identified as follows: five Astra EV implants, one Straumann implant, one Zimmer implant, and one Biomet implant. Three failed implants had never been restored, and four were supporting screw-retained provisional crowns at the time of explantation. Three implants showed early failure (after a few months) and five showed late failure (after at least one year). In the intraoral clinical evaluation, the failed implant sites presented either peri-implant erythema, suppuration, bleeding on probing (BOP), implant mobility, or a combination of these factors.

**Figure 1:**
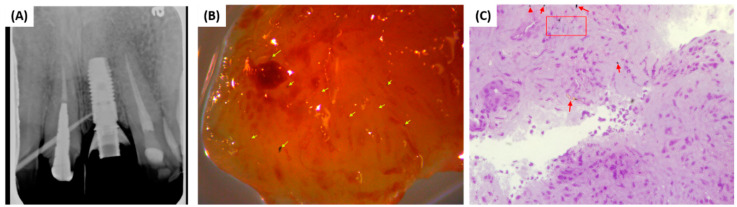
(**A**) Radiographic image of Astra EV implant; (**B**) biopsy gross sample with possible metal particles identified (yellow arrows); (**C**) possible metal particle identified (red arrows) in resin-embedded biopsy sample under optical microscope.

**Figure 2:**
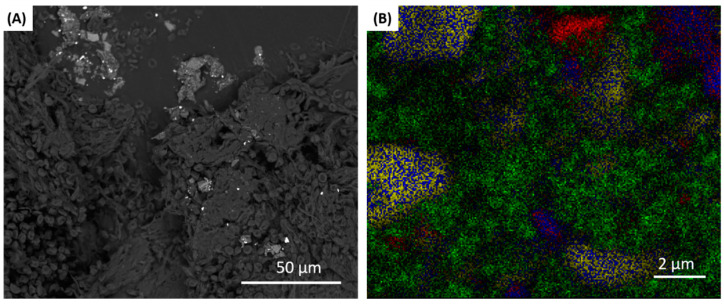
(**A**) SEM image and (**B**) elemental mapping of EDX image of biopsy: titanium (Ti) (green), calcium (Ca) (red), magnesium (Mg) (blue), and silicon (Si) (yellow).

Soft tissue biopsy and implant specimens were collected from all the participating subjects. All biopsied samples were initially viewed under an optical microscope to identify possible areas where titanium particles were located to facilitate SEM analysis (Figure 1 and Figure 2).

Radiographic evaluation prior to implant removal allowed for assessment of peri-implant bone levels (Figure 1A). The soft tissue biopsy (Figure 1B) was prepared in 500 nm thick slices for observation under the microscope (Figure 1C). If potential metal particles were identified in the biopsy sample under the microscope (Figure 1C), then the sample was further examined under SEM (Figure 2A) and EDX to conduct an elemental mapping (Figure 2B) of the elements identified in the biopsy.

Five out of eight biopsied samples revealed Ti elements in the peri-implant mucosa, suggesting that the true rate of titanium in the tissue among all failures was between 24.5% and 91.5% (the lower bound of a 95% confidence interval for the true rate of titanium presence). In addition, there were several other elements found in the biopsied samples, including Fe (iron), Cr (chromium), Si, Mg, Ca, P (phosphorus), and Al (aluminum).

**Figure 3:**
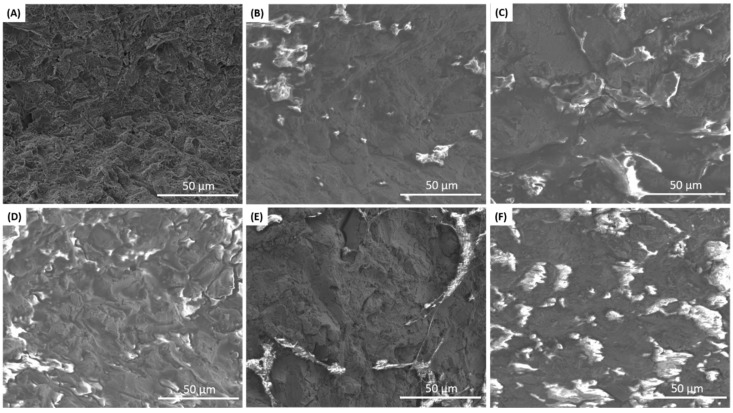
Comparison of the implant surfaces of the (**A**) reference implant versus failed implant samples (**B**) JC0480, (**C**) JS0626, (**D**) WP2173, (**E**) SS0924, and (**F**) WL4053 at the middle part of the implants.

Five out of the eight dental implant bodies collected were Astra EV implants, which are made of commercially pure titanium, grade 4. All the Astra EV implants were examined under SEM and EDX to evaluate their surface morphologies and element distributions in comparison to a reference Astra EV implant. The EDX analysis was done at various locations of the sample so that our data told an accurate story of the entire sample and any possible variations spatially. Figure 3 shows the comparison between the reference implant and the five failed Astra EV implants. The morphologies of failed implants (Figure 3B–F) seemed to be “less rough”/“less detailed” than the reference implant (Figure 3A).

Table 3 shows the weight percent of the elements on the middle surfaces of the reference implant and the sample Astra EV implants for comparison. The percentage of titanium concentration was found to be lower in most of the failed implants than in the reference implant. Samples JC0480, JS0626, and WP2173 showed titanium release in the surrounding tissue as confirmed by the tissue biopsy examination. There was a significantly lower concentration of titanium on the surfaces of the implants associated with these biopsy samples. However, samples SS0924 and WL4053 did not show titanium release in the surrounding tissue. On the one hand, sample SS0924 showed a weight percent of titanium similar to the reference implant. On the other hand, the titanium concentration in sample WL4053 was significantly lower than in the reference implant. The mean titanium surface concentration of the failed implants (55.66%) was significantly lower than the mean titanium surface concentration of the reference implant (79.2%). In addition, the elements phosphorus and calcium were found in higher concentrations in failed implants than in the reference implant.

## 4. Discussion

This study aimed to evaluate the relationship between failed implants diagnosed with peri-implantitis and the presence of Ti particles in their peri-implant soft tissues. For this purpose, eight failed implants from different subjects were identified and explanted due to clinical mobility and/or chronic infection associated with peri-implantitis. Soft tissue biopsies were also collected from the peri-implant sites and evaluated for the presence of Ti corrosion particles. Three out of the eight implants were identified to have failed within five months of placement, with two of them having been restored with provisional crowns at the 6–8 week mark. These were considered early failures. Recent studies have found that the prevalence of implant failure is higher in the early phase than in the late phase regardless of the loading time [26,27]. Even though our sample size was too small to draw conclusions on this topic, our findings of early implant failure both before and after loading are consistent with the statement above.

Five out of the eight soft tissue biopsy samples showed the presence of titanium particles, as observed histologically and confirmed with EDX examination. This suggested that the true rate among all failures was between 24.5% and 91.5% (the lower bound of a 95% confidence interval for the true rate of Ti presence). This does not necessarily mean that there was no Ti in the peri-implant sites of the other three implants, just that we were not able to identify Ti presence in the biopsied samples. The biopsies were collected from a localized area around the implant and Ti could have been present in other locations. These findings are in agreement with a study by Olmedo and co-workers [28] that detected titanium particles by EDX analysis in soft tissues associated with 10 implants explanted because of clinical mobility. Paknejad and co-workers [29] studied biopsies from 96 patients treated with two-stage approach implants by EDX and reported that all samples presented with a high density of large titanium particles. They concluded from their investigation that there was a correlation between the presence of titanium particles and the level of inflammation. However, most studies in the literature that reported the presence of titanium in peri-implant soft tissues failed to prove an association between the accumulation of titanium particles and the development of inflammation [30,31,32].

Some studies have reported the presence of other elements in the soft tissue biopsies from peri-implant sites. Wilson and co-workers [30] identified Zr (zirconia), Si, and Al, which they explained may have originated from dental cements. Tawse-Smith and co-workers [12] conducted exfoliative cytology around implants restored with zirconia abutments and identified the presence of titanium elements as well as Al, Zr, Au (gold), Ag (silver), and Cu (copper). They associated the presence of titanium with wear caused by zirconia abutments on the titanium implants; and the presence of Au, Ag, and Cu to the gold screws retaining the crowns. In our study, we identified Fe and Cr, which may be associated with the presence of nearby cast metal restorations; and Si, Mg, P, and Al, which may be associated with dental cements.

The biopsy tissue samples were collected prior to removal of the implants to prevent contamination with metal from corrosion happening at the time of implant removal. This precaution was taken given the significance of tribocorrosion, which is a material degradation process that happens as a result of the combination of friction/wear and corrosion [9]. The preferred method of implant removal in our study was the reverse torque technique, which causes the least amount of damage to surrounding tissues and to the surface of the implant [33]. However, alternative techniques were used on occasions when the reverse torque technique was not completely effective. 

SEM analysis of failed implant bodies indicated changes in surface morphology (Figure 3). The reference implant appeared rougher. The smoother surfaces on the failed implant samples could have been the result of torqueing the implants into the osteotomy at the time of placement and/or corrosion happening over time. These findings are in agreement with previous in vitro insertion torque and pullout tests by other researchers who have suggested that the processes of implant insertion and removal lead to a reduction of the oxide layer and a release of particles stripped from the surfaces [34,35]. In contrast, other authors have reported an increase in surface roughness as a result of implant placement concomitant with an increase in metal elements and debris in the surroundings [36,37,38].

Direct studies on the compositions of elements remaining on the surfaces of failed implants after explantation are scarce in the literature. Our study used control implants from the manufacturers of the failed implants to cross-examine the elemental compositions on the surfaces of the failed implants at different locations on the implant bodies. This allowed us to tell a more accurate story of the samples and account for spatial differences. Table 2 displays the results for the comparison of the Astra EV tested samples and the corresponding reference implant as an example. We found a trend for the reduction in titanium atoms and an increase in organic matter (Ca, P) on failed implants compared to the reference implant. The mean titanium surface concentration of the failed implants (55.66%) was significantly lower than the mean titanium surface concentration of the reference implant (79.2%). We also found a trend for higher presence of titanium particles in soft tissue biopsies associated with failed implants that had reduced titanium elements on their surfaces, except for sample WL4053. This failed implant, WL4053, showed a significantly lower surface concentration of titanium elements compared to the reference implant, but we did not find titanium particles in the surrounding biopsied tissue. One explanation may be that our tissue biopsy was removed from a location different from the one where the corrosion particles were located. Another explanation is that released Ti particles can be absorbed by the bloodstream and not be apparent on the peri-implant soft tissues. Several studies have suggested that titanium particles can disseminate throughout the body via the bloodstream to organs such as spleen, lung, and liver, as well as to lymph nodes [39,40,41]. Our findings of increased levels of organic matter (Ca and P) on failed implant bodies may be most likely explained by the presence of bone particles or other organic matter after retrieval from the intraoral cavity.

Four out of the five Astra EV failed implants presented clinical mobility and some degree of peri-implant bone loss. The one exception had no mobility and no bone loss but had a long history of chronic soft tissue infection that had been treated with antibiotics and surgical curettage with no success. All the patients had been previously diagnosed with either gingivitis or some form of periodontitis. All the implants showed reduced levels of titanium concentration on their surfaces when compared to the reference implant (Table 3). However, due to our small sample, definitive conclusions about the associations between clinical signs and reduced levels of implant surface titanium concentrations cannot be made at this time.

One limitation of our pilot study is the small sample size. We plan to use the data provided by this study to determine sample size for a larger study that will examine the associations between peri-implantitis, Ti corrosion, and demographic and comorbidity data. A second limitation is the lack of a control soft tissue biopsy sample from an unaffected site. There are ethical concerns in obtaining biopsy tissues in healthy peri-implant sites, as this can possibly cause disruption of the junctional epithelium and result in trauma to the implant site. A third limitation is the uneven representation of implant systems evaluated. The higher number of Astra EV failed implants found compared to other systems was because most of the implants surgically placed and restored in our specialty clinics are Astra EV.

## 5. Conclusions

This was a pilot study aimed to determine the presence of Ti in peri-implant tissues presenting signs of peri-implantitis. Ti particles were noted in five out of eight biopsied soft tissue samples. Surface corrosion was also evident on the surfaces of retrieved implant bodies, especially those associated with soft tissue containing titanium particles. To our knowledge, there has been no study that has examined Ti corrosion in peri-implant tissues and on the bodies of failed implants to this level of detail. This information adds to our base of knowledge that Ti corrosion lends a possible explanation to the progression of peri-implantitis.

## Figures and Tables

**Table 1:** Microwave settings used for biopsy.

Solution	Vacuum 20 mm Hg	Microwave Wattage	Time	Bench-Top
Fixative and washes	Yes	140	45 s	5 min
Dehydration	No	140	45 s	5 min
Resin Infiltration	Yes	140	1.5 min, paused 1 min, 1.5 min	30 min

**Table 2:** Clinical descriptive data of the samples.

Sample #	Site #	Implant Brand	Time of Survival	Restoration	Erythema	Suppuration	BOP	Mobility	Rad. Bone Loss	Implant Removal Technique	Metal Restorations Near Implant	Periodontal Condition	SRP in the Last Year	Early/Late Failure	Misch Classification
JS7069	20	Straumann	>7 years	PFM crown	No	Yes	No	No	50% bone loss, chronic infection	Reverse torque	None	Gen. mod. periodontitis	No	Late	IV. Failure
JS0626	19	Astra EV	1 yr 5 mos	Temp. crown	Yes	Yes	Yes	Yes	50% bone loss, loss osseoint.	Reverse torque	Amalgam	Gen. mild periodontitis	No	Late	IV. Failure
WP2173	9	Astra EV	2 yrs 7 mos	Temp. crown	Yes	No	No	No	No bone loss, chronic infection	Reverse torque	None	Plaque-induced gingivitis	No	Late	IV. Failure
SS0924	30	Astra EV	4 mos	Temp. crown	Yes	No	No	Yes	100% bone loss, loss osseoint.	Reverse torque, elevators	PFM crown, amalgam	Plaque-induced gingivitis	No	Early	IV. Failure
LL1153	14	Zimmer (internal hex)	>10 yrs	Unrestored	Yes	Yes	Yes	Yes	70% bone loss, loss osseoint.	Reverse torque	PFM crown, amalgam	Gen. mod. periodontitis	Unknown	Late	IV. Failure
BR5957	30	Biomet	unknown	Unrestored	Yes	Yes	Yes	Yes	60% bone loss, loss osseoint.	Trephined, elevators, forceps	Amalgam	Gen. mod. periodontitis	Yes	Late	IV. Failure
JC0480	30	Astra EV	5 mos	Unrestored	Yes	No	Yes	Yes	20% bone loss, loss osseoint.	Reverse torque	None	Loc. mild periodontitis	No	Early	IV. Failure
WL4053	5	Astra EV	5 mos	Temp. FPD	No	No	Yes	Yes	15% bone loss, loss osseoint.	Reverse torque	None	Loc. mod. periodontitis	No	Early	IV. Failure

Description of abbreviations: BOP (bleeding on probing), Rad. (radiographic), SRP (scaling and root planning), PFM (porcelain fused to metal), Gen. (generalized), Mod. (moderate), Loc. (localized), Temp. (temporary), Osseoint. (osseointegration).

**Table 3:** Comparison of the weight percent of surface elements between the Astra EV reference implant and the failed implants.

Weight Percent from Middle of Implant (Astra)						
Implant	Reference	JC0480	JS0626	WP2173	SS0924	WL4053
Oxygen	20.1	32.3	39.4	36.8	24.0	33.4
Phosphorus	0.2	1.1	4.9	3.9	0.8	6.4
Chlorine	0.2	1.3	0.1	0.1	1.4	0.5
Calcium	0.3	1.3	9.8	8.6	1.3	14.0
Titanium	79.2	63.9	45.8	50.4	72.5	45.7
Ti/(O + P + Cl + Ca)	3.81	1.77	0.85	1.02	2.64	0.84
